# Assessing the cognitive decline of people in the spectrum of AD by monitoring their activities of daily living in an IoT-enabled smart home environment: a cross-sectional pilot study

**DOI:** 10.3389/fnagi.2024.1375131

**Published:** 2024-03-28

**Authors:** Margarita Grammatikopoulou, Ioulietta Lazarou, Vasilis Alepopoulos, Lampros Mpaltadoros, Vangelis P. Oikonomou, Thanos G. Stavropoulos, Spiros Nikolopoulos, Ioannis Kompatsiaris, Magda Tsolaki

**Affiliations:** ^1^Information Technologies Institute, Centre for Research and Technology Hellas (CERTH-ITI), Thessaloniki, Greece; ^2^1st Department of Neurology, G.H. “AHEPA”, School of Medicine, Faculty of Health Sciences, Aristotle University of Thessaloniki (AUTH), Thessaloniki, Greece; ^3^Greek Association of Alzheimer’s Disease and Related Disorders (GAADRD), Thessaloniki, Greece; ^4^Laboratory of Neurodegenerative Diseases, Center for Interdisciplinary Research and Innovation (CIRI - AUTh), Balkan Center, Buildings A & B, Aristotle University of Thessaloniki, Thessaloniki, Greece

**Keywords:** Alzheimer’s disease, healthy controls, subjective cognitive decline, mild cognitive impairment, smart home, sensor technology, activities of daily living

## Abstract

**Introduction:**

Assessing functional decline related to activities of daily living (ADLs) is deemed significant for the early diagnosis of dementia. As current assessment methods for ADLs often lack the ability to capture subtle changes, technology-based approaches are perceived as advantageous. Specifically, digital biomarkers are emerging, offering a promising avenue for research, as they allow unobtrusive and objective monitoring.

**Methods:**

A study was conducted with the involvement of 36 participants assigned to three known groups (Healthy Controls, participants with Subjective Cognitive Decline and participants with Mild Cognitive Impairment). Participants visited the CERTH-IT Smart Home, an environment that simulates a fully functional residence, and were asked to follow a protocol describing different ADL Tasks (namely Task 1 – Meal, Task 2 – Beverage and Task 3 – Snack Preparation). By utilizing data from fixed in-home sensors installed in the Smart Home, the identification of the performed Tasks and their derived features was explored through the developed CARL platform. Furthermore, differences between groups were investigated. Finally, overall feasibility and study satisfaction were evaluated.

**Results:**

The composition of the ADLs was attainable, and differentiation among the HC group compared to the SCD and the MCI groups considering the feature “Activity Duration” in Task 1 – Meal Preparation was possible, while no difference could be noted between the SCD and the MCI groups.

**Discussion:**

This ecologically valid study was determined as feasible, with participants expressing positive feedback. The findings additionally reinforce the interest and need to include people in preclinical stages of dementia in research to further evolve and develop clinically relevant digital biomarkers.

## 1 Introduction

According to the World Health Organisation (WHO), there are currently over 55 million people living with dementia (PwD) globally (World Health Organisation Dementia Key Facts, 2022). The sharp increase in dementia cases is likely to have significant consequences for healthcare providers, caregivers, and the economy (Aranda et al., 2021). For this, research has focused on the early detection of dementia with the primary objective to intervene before symptoms worsen and lead to loss of independence and greater need for care (Rasmussen and Langerman, 2019).

This is further supported by the fact that search for effective treatments of AD has led to the first disease-modifying therapies (Lecanemab and Aducanumab). These treatments have been approved by the FDA as well as in Japan and are being considered by the EMA (European Medicines Agency, 2023). Furthermore, 141 drugs are currently being tested in clinical trials for the treatment of AD, 80% of which aim to slow disease progression (Cummings et al., 2023).

The need to identify people at the pre-symptomatic stage becomes eminent, as the recently developed therapeutic agents exhibit their greatest potential in early AD (Cummings et al., 2023; van Dyck et al., 2023). Additionally, lifestyle and other non-pharmacological interventions (e.g., the multidomain FINGER intervention (Ngandu et al., 2022)), show promising results in preventing symptom progression when applied timely, before the onset of dementia.

An early sign of dementia is functional deterioration expressed often through difficulties in performing Activities of Daily Living (ADLs), as an association has been found to exist between ADL deficits and cognitive functioning (Bangen et al., 2010; Jekel et al., 2015). Current approaches for assessing ADLs to determine functional decline involve traditional pen and paper methods. As these rely on informant input and are often not sensitive enough to capture subtle changes, there is further space for improvement and development of complementary measures (Sikkes et al., 2009).

Shifting focus to unobtrusive, passive, objective monitoring approaches, digital biomarkers have emerged showing promising potential (Anna-Katharine et al., 2023). In a general sense, using technology-based approaches to evaluate ADLs in older adults is a promising area of research with several advantages over traditional cognitive assessment methods. However, a major drawback of these tools is that they may require prolonged use to detect subtle ADL differences that indicate cognitive decline. Nevertheless, the obtained information from digital biomarkers, reflect real-life conditions, while eliminating reporting bias. They can be derived from passive sensors, wearables, purposive technological solutions (e.g., games) and other technological solutions (e.g., assessment of computer mouse movements, identify if pill box used) (Piau et al., 2019). Digital biomarkers can be used to assess walking and sleep patterns, physical activity and also, ADLs. They represent a valuable method, as they comprise sensitive and precise measures that can detect subtle changes. This makes them suitable in assessing deterioration in function that can occur at an early, preclinical stage.

A plethora of sensors has been used and deployed in the context of Smart Homes (SH) (in the sense of controlled research environments, care homes or participants’ homes where the sensors are being installed) allowing for remote in-home sensing and remote ADL monitoring (Garcia-Constantino et al., 2021; Moyle et al., 2021). There are many opportunities for the use of various SH technologies in community-dwelling PwD, ranging from diagnostic assessment to long-term and personalized care management. As a result, many individual studies have been conducted on the development and use of SH technologies in older populations (Ma et al., 2023; Yu et al., 2023). Such technologies are being investigated for use in a wide range of applications and contexts. These can vary from home based monitoring, personalized care, quality of life improvement, to independent living, observation and prediction of the actions of a person, caregiver burden reduction, intervention and disease progression monitoring, and also identification of emergency situations (Amiribesheli and Bouchachia, 2018; Ault et al., 2020; Han et al., 2022; Miller et al., 2022). Furthermore, there is a growing interest in the use of using digital biomarkers assessing ADLs, as reliable proxies for screening participants for clinical trials or as secondary endpoints (Atkins et al., 2015; Gold et al., 2018).

The use of sensor technology to identify cognitive decline through observing ADL performance is not a novel concept. Even so, the field of exploring methods and developing digital biomarkers to quantify and compare ADL performance is still in its infancy.

### 1.1 Aim of the present work

This work has been conducted in the context of RADAR-AD,^1^ an EU-funded project that explores the potential of mobile and digital technologies to improve the assessment of Alzheimer’s Disease (AD) (Owens et al., 2020; Muurling et al., 2021). In particular, the main motivation in one of RADAR-AD’s sub-studies was to explore whether the identification and monitoring of ADLs was achievable, utilizing data collected from in-home sensors in a Smart Home environment. Furthermore, it was investigated if the identified ADLs can provide clinically meaningful insights regarding the preclinical stages of AD. Additionally, technology acceptance and the overall feasibility of the study was assessed.

**Figure 1:**
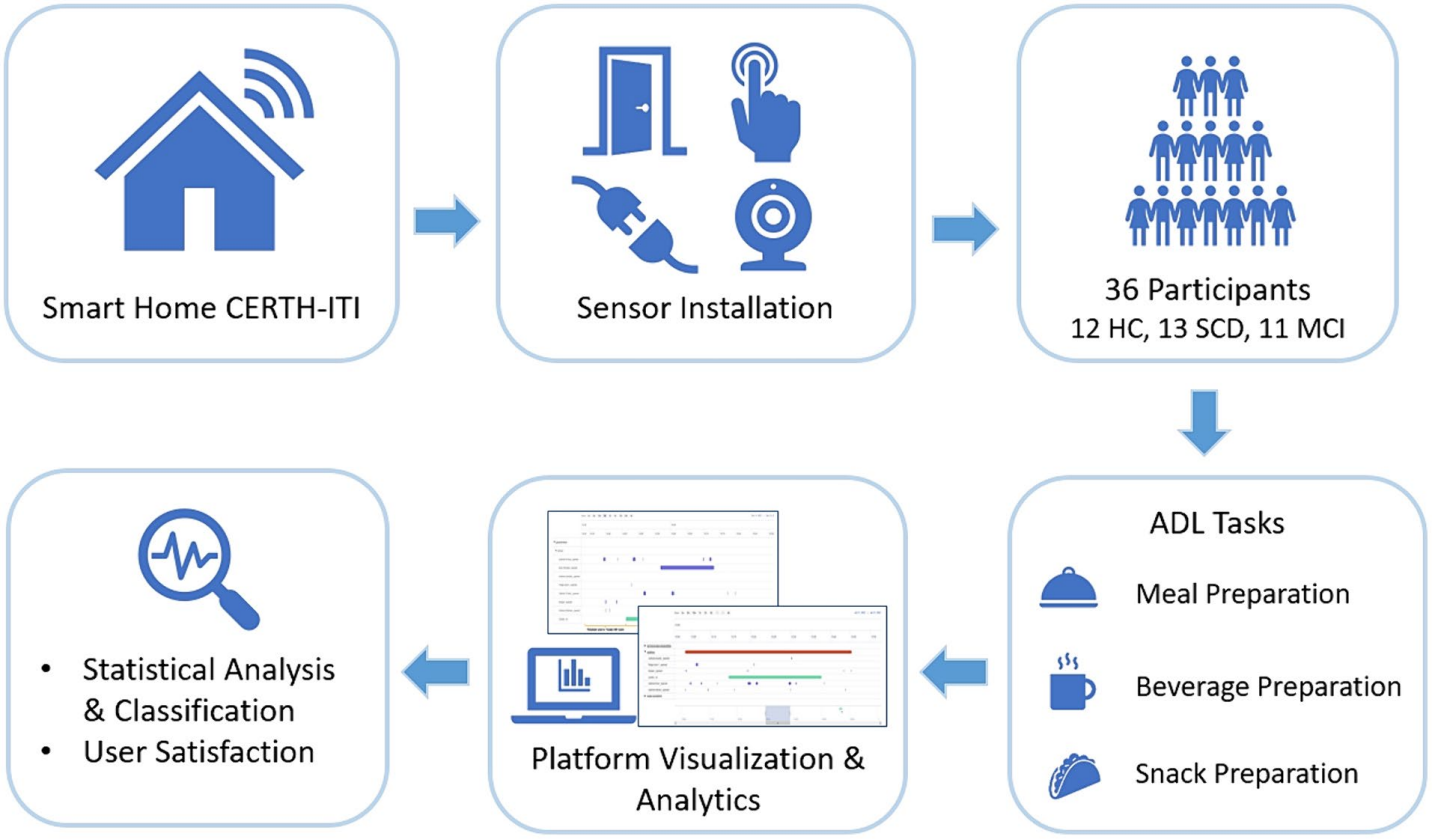
Study pipeline.

In detail, we assessed a number of people at preclinical and prodromal stages of AD, namely, the Subjective Cognitive Decline (SCD) stage, and the Mild Cognitive Impairment (MCI) stage (Dubois et al., 2016), that were evaluated against healthy control (HC) participants in terms of their performance during the execution of particular ADLs. Their performance was monitored through the data collected by a set of commercially available fixed in-home sensors^2^ installed in CERTH-ITI’s Smart Home.^3^ The sensor data were collected, processed and visualized using a platform developed by our research team (Mpaltadoros et al., 2021). First insights could be gained, regarding the effectiveness of remotely monitoring ADLs and their potential to offer quantifiable metrics for discriminating between the different stages of cognitive impairment. Furthermore, all participants filled a detailed questionnaire assessing overall study satisfaction while staying at CERTH-ITI’s SH, evaluating the presented sensor technologies. The study pipeline is given in Figure 1.

## 2 Materials and methods

### 2.1 Study protocol

#### 2.1.1 Participants

Participants were recruited from the Greek Association of Alzheimer’s Disease and Related Disorders (GAADRD)^4^ and a wide community audience. The study was carried out in accordance with the Declaration of Helsinki and received approval by the Ethics Committee of CERTH (ETH.COM 54/17-06-2020) and the Scientific and Ethics Committee of GAADRD (242/2022 ΑΙ_07/10/2021), while a written informed consent was obtained from all participants prior to their participation in the study. The Information Forms used to debrief the participants were prepared according to ICH-GCP requirements and data protection regulations [European Medicines Agency (EMA), 2016].

The diagnosis of HC, SCD and MCI was set by a neuropsychiatrist, specialised in dementia, according to the structural magnetic resonance imaging (MRI), medical history, neuropsychological tests and neurological examination. The MCI group fulfilled the Petersen criteria (Petersen et al., 2009) and it is noted that all MCI cases were of the amnestic subtype. The SCD group met IWG-2 Guidelines (Dubois et al., 2014) as well as the SCD-I Working Group instructions (Molinuevo et al., 2017). Regarding the SCD and MCI groups, we excluded participants with confounding factors based on blood tests (hormonal disorders, vitamin deficiency etc.), while structural MRI scans were done for participants in both groups (vascular/demyelinating lesions, tumours, anatomical variations etc.). Additional inclusion criteria for the SCD and HC participants included having a normal general medical, neurological and neuropsychological examination. Exclusion criteria comprised severe psychiatric, physical or other neurological disorder, illness or any other somatic disorder, which may cause cognitive impairment. Additionally, it is noted that as the study protocol included an EEG based action [explored in Ioulietta et al., (2023)], left-handedness constituted an exclusion criterion (Patel and Azzam, 2005; Cuzzocreo et al., 2009).

In total, forty participants were recruited, of whom two participants were considered drop-outs, while data from two participants were removed from the analysis to ensure that the groups were age-matched, leading to a total of thirty six participants (*N* = 36). In detail, the HC group consisted of 12 participants, the SCD group of 13 and the MCI group included 11 participants. The demographic characteristics of the participants can be found in Table 1. All groups exhibited a similar range of age and education. Kruskal-Wallis test revealed no group differences with regards to age and years of education (Table 1).

**Table 1:** Demographic characteristics of the participants (*N* = 36).

	HC	SCD	MCI	*p*	*N* = 12	*N* = 13	*N* = 11
*Demographic characteristics*				
Age in years	63.9 (6.4)	64.4 (6.4)	69.7 (6.4)	0.109
Gender (F:M)	11:1	9:4	8:3	
Years of education	13.8 (2.6)	14.6 (2.1)	12.9 (2.7)	0.292
*Neuropsychological tests*				
Mini Mental State Examination (MMSE) (Folstein et al., 1975)	29.25	27.85	26.00	**<0.001**
Montreal Cognitive Assessment (MoCA) (Nasreddine et al., 2005)	26.83	25.54	20.64	**<0.001**
Functional rating scale for symptoms of dementia (FRSSD) Total Score (Hutton et al., 1998)	2.25	2.62	3.27	0.181
Functional and Cognitive Assessment Test (FUCAS) Total Score (Kounti et al., 2006)	42.00	42.00	44.36	**<0.001**
Rey-Osterrieth Complex Figure Test (ROCFT) Copy (Osterrieth, 1944)	35.25	33.00	30.23	**<0.001**
Rey-Osterrieth Complex Figure (ROCFT) Delayed Recall (Osterrieth, 1944)	18.50	20.19	10.86	**0.002**
Rivermead Behavioral Memory Test (RBMT) Immediate Recall (Wilson et al., 1989)	15.42	13.85	10.45	**0.003**
Rivermead Behavioral Memory Test (RBMT) Delayed Recall (Wilson et al., 1989)	13.83	11.96	7.55	**0.002**
Rey Auditory Verbal Learning Test (RAVLT) Total Score (Rey, 1964)	45.17	39.15	34.00	**0.025**
Trail Making Test (TMT) Part B (Tombaugh, 2004)	146.67	151.38	217.82	**0.045**
Verbal Fluency Test (FAS) (Kosmidis et al., 2004)	11.44	10.13	9.43	**0.009**
Alzheimer’s Disease Assessment Scale–Cognitive Subscale (ADAS-Cog) (Rosen et al., 1984)	9.47	11.96	16.58	**0.001**

Bold values denote statistical significance at the *p* < 0.05 or *p* < 0.001 level.

As the study was conducted during the pandemic (2021), solely fully vaccinated (validated vaccination certificates with verified app) participants were recruited. Moreover, after each participant’s visit decontamination by experts took place at the SH to ensure the safety of all people involved.

#### 2.1.2 Study design

**Figure 2:**
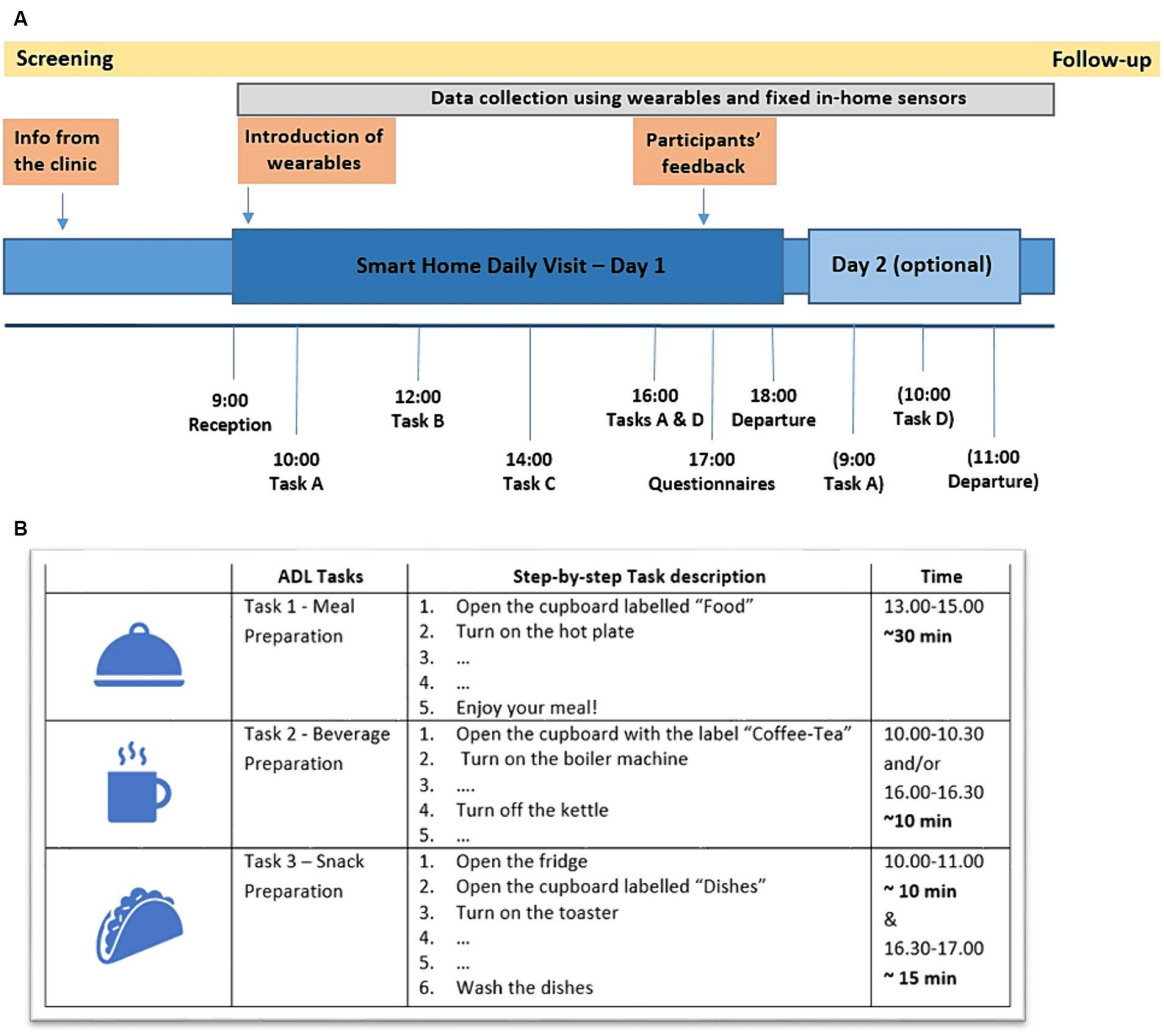
**(A)** Overview of the complete study protocol and the structure of the daily visit with the optional overnight stay. (Task A – Beverage Preparation, Task B – Physical Exercise and Meditation Sessions, Task C – Meal Preparation, Task D – Snack Preparation). **(B)** Overview of the activities protocol, describing step-by-step the Tasks to be performed during the participants’ visit (the full step-by-step description can be found in the Supplementary information).

Participants had the option of staying overnight at the SH or only for a daily visit. The study protocol consisted of five Tasks, of which three Tasks comprised ADL activities, namely, Task 1- Meal Preparation, Task 2 - Beverage Preparation and Task 3 - Snack Preparation (Figure 2). Two tasks consisting of meditation sessions were also included in the protocol (Task 4 - Mindfulness Based Stress Reduction –MBSR; Crane et al., 2017; Creswell et al., 2019), and Task-5 Kirtan Kriya meditation (Khalsa, 2015), where participants’ performance during meditation was monitored using a portable Muse EEG device. The protocol and the study outline have been presented in Stavropoulos et al. (2021a) and Lazarou et al. (2022) while the results of the meditation sessions have been reported in a separate publication (Ioulietta et al., 2023). The complete protocol and the full step by step description of each Task, as given to the participants, can be found in the Supplementary information. The total duration of the study (visit of first participant until visit of last participant) was approximately 3 months.

Upon arrival, participants were welcomed to the SH by the researchers and a detailed tour of the house followed. Afterwards, time for discussion and additional questions was planned and the study structure/protocol was again presented to the participants. Researchers then left the SH, and participants were encouraged to feel at home and perform the requested ADLs alone. For emergencies, they could contact the researchers via telephone or press one of the installed panic buttons. A psychologist- clinical research associate at CERTH was at all times available.

#### 2.1.3 Participants’ feedback (feasibility assessment)

At the end of the visit, questionnaires regarding study feasibility and technology evaluation were distributed to the participants, namely an overall study satisfaction questionnaire, the System Usability Scale (SUS), and the PANAS questionnaire assessing positive and negative affect (Supplementary information; Brooke, 1986; Watson et al., 1988).

### 2.2 Infrastructure

#### 2.2.1 Smart home setting

**Figure 3:**
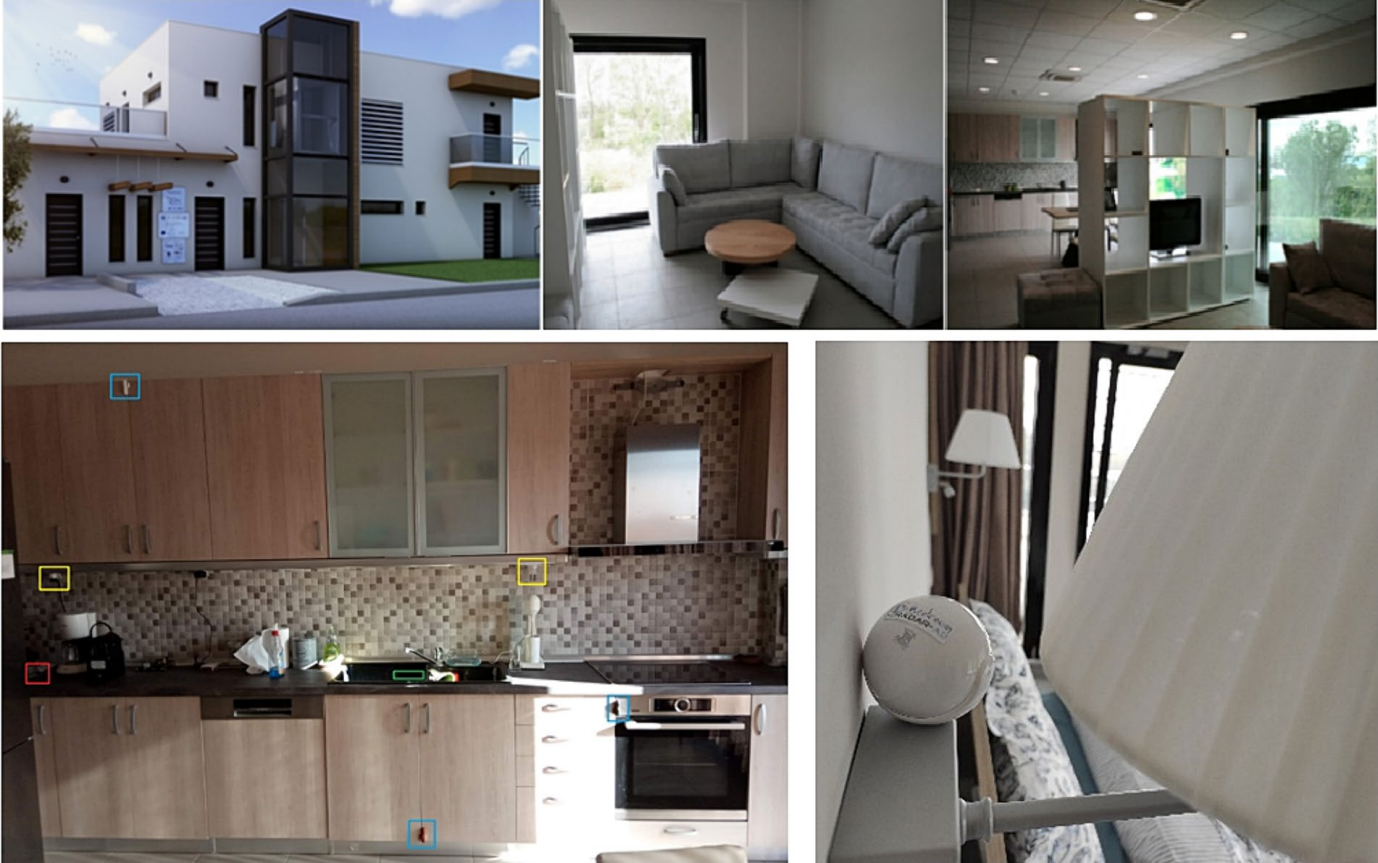
Top: The smart home site. Bottom: Examples from the sensor installation in the smart home (left: marked Wall Plugs, Door/Cabinet and motion sensors in the kitchen, right: Motion sensor in the bedroom).

The study was performed in the CERTH/ITI nZEB SH (Figure 3), a fully equipped, real domestic building, where participants can engage in real-world living scenarios and explore a plethora of innovative, smart IoT-based technologies. The SH can be used to test, validate and evaluate novel technologies from various fields, including but not limited to, Health, Energy, Big Data, Robotics and Artificial Intelligence (AI).

In this study, the SH environment was used to resemble and simulate the participants’ home, with the installation of a number of sensors in every room allowing for unobtrusive monitoring of participants’ ADLs. The available to the participants’ spaces in the SH included one living room, a kitchen, a bedroom and two bathrooms.

#### 2.2.2 IoT devices infrastructure

##### 2.2.2.1 Installed sensors

IoT device selection resulted from extensive literature research and discussions with the partners of the RADAR-AD Consortium^5^ (Owens et al., 2020; Stavropoulos et al., 2020). Also, focus groups with EWGPWD^6^ and Alzheimer Europe^7^ were assembled in order to rate the devices based on their features and potential usage and finalize the selection process (Stavropoulos et al., 2021b). Furthermore, an online meeting was organized (11/03/2021) to collect the EWGPWD’s feedback on the fixed in-home sensors used in this study.

For the present study, commercially available Motion Sensors (quantity, *n* = 8) were added in every room of the SH to detect human presence. Furthermore, Door/Cabinet Sensors (*n* = 8) were placed on the main doors, as well as on the kitchen cabinets and drawers to signal if they were being opened and closed. Wall Plugs (*n* = 6) to measure consumption were added to small electrical appliances (e.g., kettle, toaster, hot plate) and four panic buttons were placed in the SH for emergencies. Examples of the installed fixed in-home sensors can be seen in Figure 3.

##### 2.2.2.2 Raw data

The sensors generate data that consist of two types of time series, Signals, and Consumptions, both of which express the change of a device’s status or metrics, respectively. Motion, Door/Cabinet sensors and Panic Buttons compose the Event time series, expressing with Boolean values the sensor’s status (1 for Activated and 0 for Idle). The sensors are activated when a person interacts with them or with the environment (e.g., Entering a room activates the Motion Sensor, Opening the Cupboard activates the Cupboard’s sensor, pressing the Panic Button sends the corresponding signal). Wall Plugs on the other hand, express the change of a home appliance’s power consumption.

##### 2.2.2.3 Hubs

The time series are generated via a small gateway device^8^ designed to manage an entire SH system. Signal time series comprise signals from all sensors except for Wall Plugs, for which a Consumption time series is generated separately. In addition, the gateway device provides a REST API to serve the data to other services, such as the CARL Platform developed by our research team (Mpaltadoros et al., 2021).

#### 2.2.3 Data collection and visualization

##### 2.2.3.1 Data model

The CARL Platform (Care Ally: Data Collection and Analysis Platform for Assisted Living) is an end-to-end data collection and analysis platform that allows integration with a continuously expandable list of commercially available wearable and IoT sensors and apps. Additionally, the platform offers a Visualization Dashboard for clinicians (real time data representation), to enable operational and clinical oversight across the entire lifespan of a study, in this way facilitating informed decision-making.

Integration of the gateway with the CARL Platform was achieved with the development of two components, the CARL RPi Client and an Adapter. The CARL RPi Client is a client service designed to detect the gateway on a local network and consume the generated time series in order to upload them to the CARL Platform. The Adapter was responsible for the authentication of the incoming data from CARL RPi Client instances and the serialization of the raw data. In this way, all data was transferred to the CARL Platform central database.

##### 2.2.3.2 Visualization services

**Figure 4:**
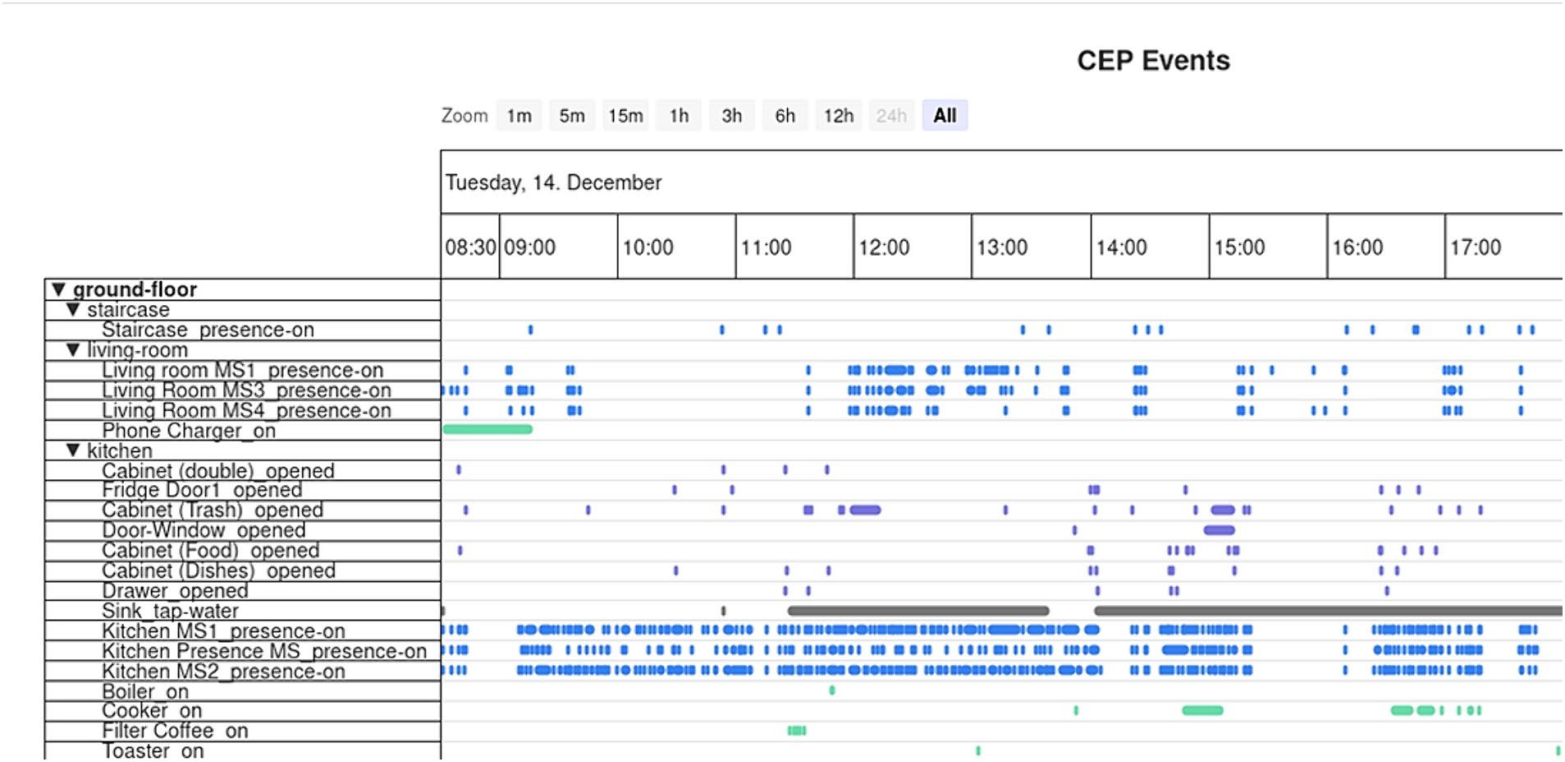
Event objects visualization using the CARL platform visualization dashboard.

Once the raw data was saved in CARL Platform’s database, it was processed to produce Event Objects, representing the duration of various events that occurred during the participant’s visit (e.g., Cupboard Opened, Kitchen Presence, Hot Plate On). A clinician could then visualize these Event Objects through the dashboard, gaining an overview of all the participant’s interactions with the environment (Figure 4).

### 2.3 Monitoring and synthesis of ADLs

#### 2.3.1 From raw data to ADLs

**Figure 5:**
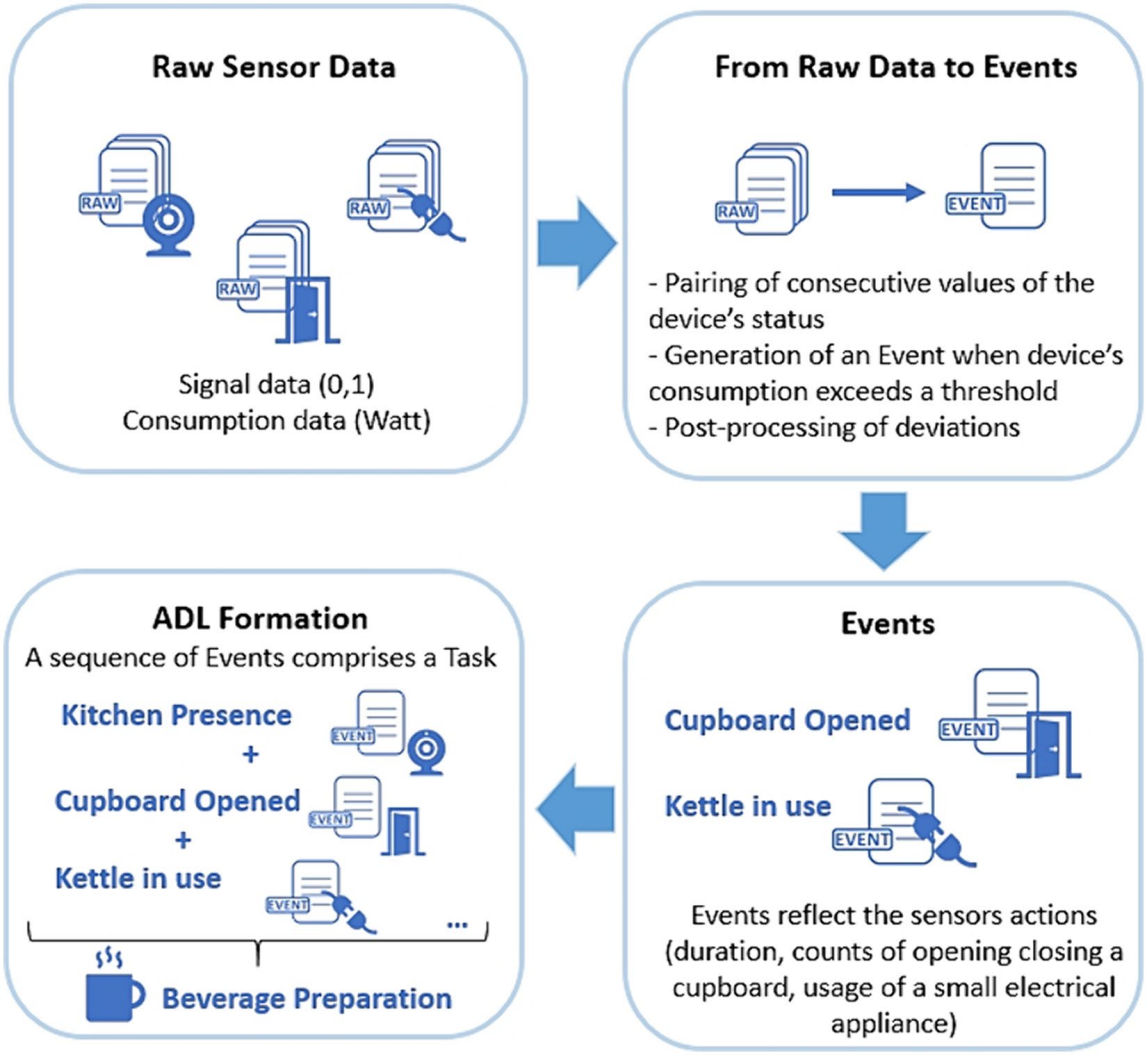
An overview of the transformation of raw sensor data into ADLs.

In this section, a detailed description of the process followed to structure and transform the raw data into Tasks and ADLs is presented. An overview is given in Figure 5, where it can be seen how the raw signal and consumption data are converted to Events, while sequences of these Events are utilized to form ADLs.

##### 2.3.1.1 Raw data to events

The production of Event objects based on Signal data is achieved by pairing the consecutive alterations of the device’s status. When a Signal has a “newValue” of 1, a new Event object is generated, having as starting point the Signal’s timestamp. The next Signal with a “newValue” of 0, will act as the ending point of the Event. An overview can be found in Table 2.

**Table 2:** Signal information obtained by the sensors (CARL: Care Ally: data collection and analysis platform for assisted living).

Signal information	Explanation
Id	Unique identifier
Timestamp	When it occurred in unix epoch
deviceID	Device’s unique identifier
deviceType	Device’s type
oldValue	Previous status
newValue	Current status

##### 2.3.1.2 Raw consumption data to event

For Consumption based Events, we took into consideration that all devices, even when idle, still consume electrical power. Therefore, depending on the home appliance, we applied an empirical threshold, used to define when the home appliance was turned on and off. If the consumption value exceeded the set threshold, then an Event object was generated with the start time equivalent to the Consumption’s timestamp. The next Consumption’s timestamp with a value below the threshold, was used to mark the Event Object’s end time.

##### 2.3.1.3 Post-processing deviations

Due to the nature of the Cupboard/Door sensors, deviations were noticed in the duration of some related events (e.g., the cupboard did not fully close due to the brakes and extreme values were captured). On such occasions, the events were post-processed by inspecting each participant’s Event objects from the sensor, to determine the distribution of the values. The duration values at the 75th percentile were then compared to the sum of the 75th percentile and Standard Deviation values. If the duration value exceeded the sum, we updated the end time of the Event to match the 75th percentile value. This process was applied until all duration values were lower than the sum value.

##### 2.3.1.4 Events to ADLs

Through the CARL platform, it is possible to check whether the sensors were successfully activated by the participants compared to the task descriptions provided. An example of a step by step description and the respective expected sensor activations are presented in Table 3 for Task - 2 Beverage Preparation.

**Table 3:** Example of an ADL and the respective sensor activation sequence.

Example – Step by step description for Task 2 - beverage preparation	Sensors activated
In the kitchen, fill the kettle with water and turn it on from the button Open the cabinet labelled “Coffee - Tea” … Make sure you close the button from the kettle After finishing drinking your coffee, wash the cup and the kettle with the dish sponge and leave them in the sink to dry	Motion Sensor “Kitchen Presence” (ON) Wall plug sensor “Kettle” (ON) Door/Cabinet sensor “Coffee/Tea Cabinet” ON for a second then OFF … Wall Plug sensor “Kettle” (OFF) Motion Sensor “Kitchen Presence” (OFF)

In order to form each of the three ADL Tasks, the use of a small electric appliance, depending on the task was considered necessary. In detail, for Task 1 – Meal Preparation the hot plate should be used, in Task 2 – Beverage Preparation the kettle was needed, while in Task 3 – Snack Preparation, the toaster was considered essential.

**Figure 6:**
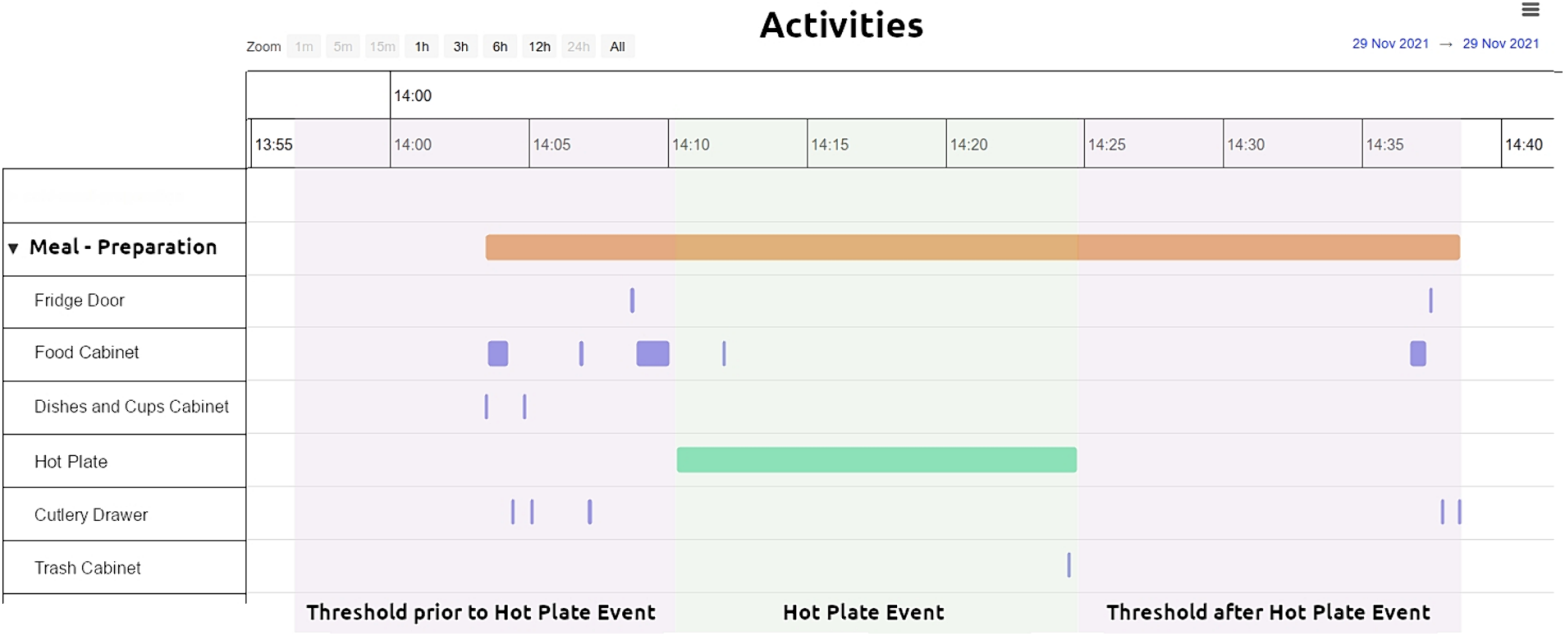
ADLs based on the collected data - threshold applied to small electrical appliances consumption (e.g., hot plate). Task – 1 Meal Preparation activity visualized through the CARL platform.

In Figure 6 the rationale of forming an ADL (Task 1 – Meal Preparation) is given. Initially, a home appliance based event (“Hot Plate Event” green bar, Figure 6) was detected. In order to take into account the event, its duration had to exceed a specific value. This was set by the researchers during the testing phase and served as a checkpoint (minimum duration for the hot plate *t* = 10 min, kettle *t* = 2 min and toaster *t* = 5 min). From there, thresholds were applied before and after the appliance’s related event (“Threshold prior to Hot Plate event” and “Threshold after Hot Plate Event” Figure 6). The thresholds were determined after manual inspection of the data of all participants and were set for the hot plate at *t* = 15 min, the kettle at *t* = 5 min and the toaster at *t* = 5 min. All relevant Event objects occurring in between these thresholds (purple bars and lines, green bar, Figure 6) were clustered into one entity leading to an ADL (orange bar, Figure 6).

Apart from allowing the formation of the ADLs, visualization through the CARL platform offers a plethora of information on the performed tasks. For example, for Task 1 – Meal Preparation (Figure 6) it can be seen in which order the different sensors were activated and for how long during a specific point in time, while the participant performed the task. In detail, the orange bar shows the duration of the complete Task, information derived by considering all the individual sensors involved in the ADL performance as described above. The green bar shows the duration of the electrical appliance in use, in this case the hot plate, derived by the consumption observed during this period of time. The more frequent (due to number of repetitions) and thinner (due to shorter duration) purple signals, show the various cabinets and drawers opened and closed during the execution of the Task.

#### 2.3.2 ADL features

Our intention with analysing the raw sensor data into events and ADLs associated with specific Tasks, was to enable the extraction of representative features characterising an ADL, and use these features to detect differences between the groups of participants (HC, SCD and MCI).

More specifically, we can see the time of the day the participants performed each task, the duration of each activity (in seconds), as well as the duration each appliance was in use (in seconds), or the time a cabinet was left open, and the number of times a sensor was activated (number of repetitions, count for, e.g., opening a cabinet). Furthermore, apart from these primary derived features, a secondary feature/by-product was investigated, namely the “Inaction Time” which refers to the time recorded between sensor activation. For this, the durations between different sensor signals were added up and subtracted from the total activity duration creating the feature “Inaction Time.”

In Table 4 the description of each feature is given, along with the naming convention followed for each sensor, the sensor type and in which of the Tasks they were utilized. All this information was exported in the form of a csv file, to facilitate statistical analysis.

**Table 4:** Feature description and the naming convention followed for the sensors used in each Task/ADL.

Feature	Description		Data type
Activity_name	ADL’s Name (i.e., Task 1 – Meal Preparation, Task 2 – Beverage Preparation, Task 3 – Snack Preparation)		Text
Activity_duration	The time needed (duration) to perform an ADL (Task 1, Task 2, Task 3). All sensors comprising the ADL are taken into account [The time between the start_time of the first Event Object and the end_time of the last Event Object]		Seconds
Number_of_steps	The total number of sensors activated during the performance of an ADL		Integer
Count_ < name_of_sensor>	The number of times, number of repetitions (count) a Door/Cabinet sensor was activated		Integer
Sum_ < name_of_sensor>	The time (SUM duration) of a sensor being activated		Seconds
Avg_ < name_of_sensor>	The time (AVG duration) of a sensor being activated [SUM duration divided by the number of repetitions (sum duration and average duration are identical if sensor was used/activated once)] [sum_ < name_of_sensor>/count_ < name_of_sensor>]		Seconds
Sum_ < inaction_time>	Time period during a Task where the participant did not activate any sensors [activity_duration – SUM (sum_ < sensors>)]		Seconds
**Sensor name**	**Type of sensor**	**Sensor description**	**Task**
Coffee – Tea cabinet	Door/cabinet sensor	Door/drawer/cabinet opening - closing	2
Dishes and cups cabinet			1, 2, 3
Cutlery drawer			1, 2, 3
Food cabinet			1, 3
Trash cabinet			1, 2, 3
Fridge door			1, 3
Hot plate	Wall plug	Consumption monitoring	1
Kettle			2
Toaster			3
Kitchen, living room, bedroom, bathrooms, hallways	Motion sensors	Presence/motion capture	ADLs were performed in the Kitchen

#### 2.3.3 Validation

To ensure the sensors’ and platform’s effectiveness and reliability, during the study, information was gathered from the participants by the researchers in the form of free text notes, regarding the performed ADLs (completed Tasks, approximate time of the day performed) and used as ground truth. A comparison between the ground truth and the activities identified by the platform was made. Differences in the number of activities recorded by the platform and the available ground truth data could be attributed to power and internet outage or sensor connectivity issues. In detail, one “Meal Preparation” and one “Beverage Preparation” tasks were missing from the platform due to unexpected power outage in the SH. For two “Snack Preparation” tasks (performed the same day), the platform collected only data from the Wall Plug sensor, while the Door/Cabinet sensors were unresponsive.

#### 2.3.4 Statistical analysis

With the dataset containing all information on the various features per task at hand, we proceeded to compare the performance on each ADL, among the three groups at the level of significance *p* = 0.05. Descriptive analysis and statistical analysis were performed using SPSS v25.0 for Windows (IBM Corporation, Armonk, NY, United States). Descriptive analysis was performed to depict participants’ data, while statistical analysis was carried out to locate differences in the various activities and the individual features.

For assessing the normality assumption for continuous variables we used the Kolmogorov–Smirnov test. As the depended variables were not normally distributed, and due to the small sample size available, non-parametric tests were selected (Mishra et al., 2019). Between groups comparisons were made using the Kruskal-Wallis test. For examining the potential statistical significance between two independent groups (e.g., HC versus SCD), the Mann–Whitney test was used. Furthermore, the Area under the Curve (AUC) was also examined.

## 3 Results

### 3.1 Exploring ADLs – task comparison between groups

The assumptions formed in the present study were shaped around the expectation that more cognitively impaired participants will exhibit different behavioural patterns compared to healthy controls. These differences can be attributed to functional deterioration, as AD is characterized by the impairment of cognitive functions and increasingly poorer ADL performance.

Specifically, it is expected that the differences in ADL performance will be observed in the overall time needed to complete an ADL, in additional steps made and repeated actions noted (e.g., opening/closing a cabinet more frequently).

Consequently, the features considered meaningful to explore these assumptions, as derived from feature engineering of the collected sensor data, include number of steps to complete an ADL, activity duration, sensor activation duration, number of sensor activations, and inaction time.

Descriptive statistics and results for the statistical tests are given for all features in the Supplementary information. It is noted that while all results are commented in the text, only the more prominent for discussion features are presented in Figures and Tables to provide the reader with a clearer overview.

#### 3.1.1 Task completion

Participants were asked to complete three Tasks as entailed in the protocol. Three tasks were completed by 33% of the participants of the HC group (4/12), 23% of the SCD (3/13) and 18% (2/11) of the MCI group. Two tasks were performed by the majority of the SCD group (61.5%, 8/13), approximately half the participants of the MCI group (55%, 6/11) and by 42% (5/12) of the HC group. Furthermore, 27% (3/11) of the MCI group completed only one task, whereas the percentages are 25% (3/12) and 15% (2/13) for the HC and the SCD group, respectively.

In detail, it is noted that 11/12 HC (91.67%), 11/13 SCD (92.31%) and 7/11 MCI (63.64%) performed the activity “Meal Preparation.” The activity “Beverage Preparation” was performed by 10/12 HC (76.92%), 11/13 SCD (92.85%) and 6/11 of the MCI (61.53%). Only twelve participants performed the Task “Snack Preparation,” in detail, 5/12 HC, 4/13 SCD and 3/11 MCI.

#### 3.1.2 Number of steps

The estimated number of steps needed to complete Task 1 – Meal Preparation, according to the step-by-step task description is ten. The mean number of steps for each group was found to be 14.7 (SD = 3.8) for HC, 14.6 (SD = 5.6) for SCD and 18.8 (SD = 8.2) for the MCI group, showing no differences between the HC and SCD groups, and a larger number of steps for the MCI group. No statistical significance was noted (Kruskal-Wallis test *p* = 0.437).

For Task 2 - Beverage Preparation, the description included six sensor activation steps, while participants performed HC = 7 (SD = 1.5), SCD = 7.1 (SD = 1.3), MCI = 7.6 (SD = 3.9) steps.

The three groups needed approximately the same number of mean steps to complete Task 3 – Snack Preparation [HC = 9.8 (SD = 2.9), SCD = 11.3 (SD = 4.9), MCI = 10.0 (SD = 3.6)]. It is noted that the protocol lists six sensor activation steps for this Task.

#### 3.1.3 Activity duration

**Figure 7:**
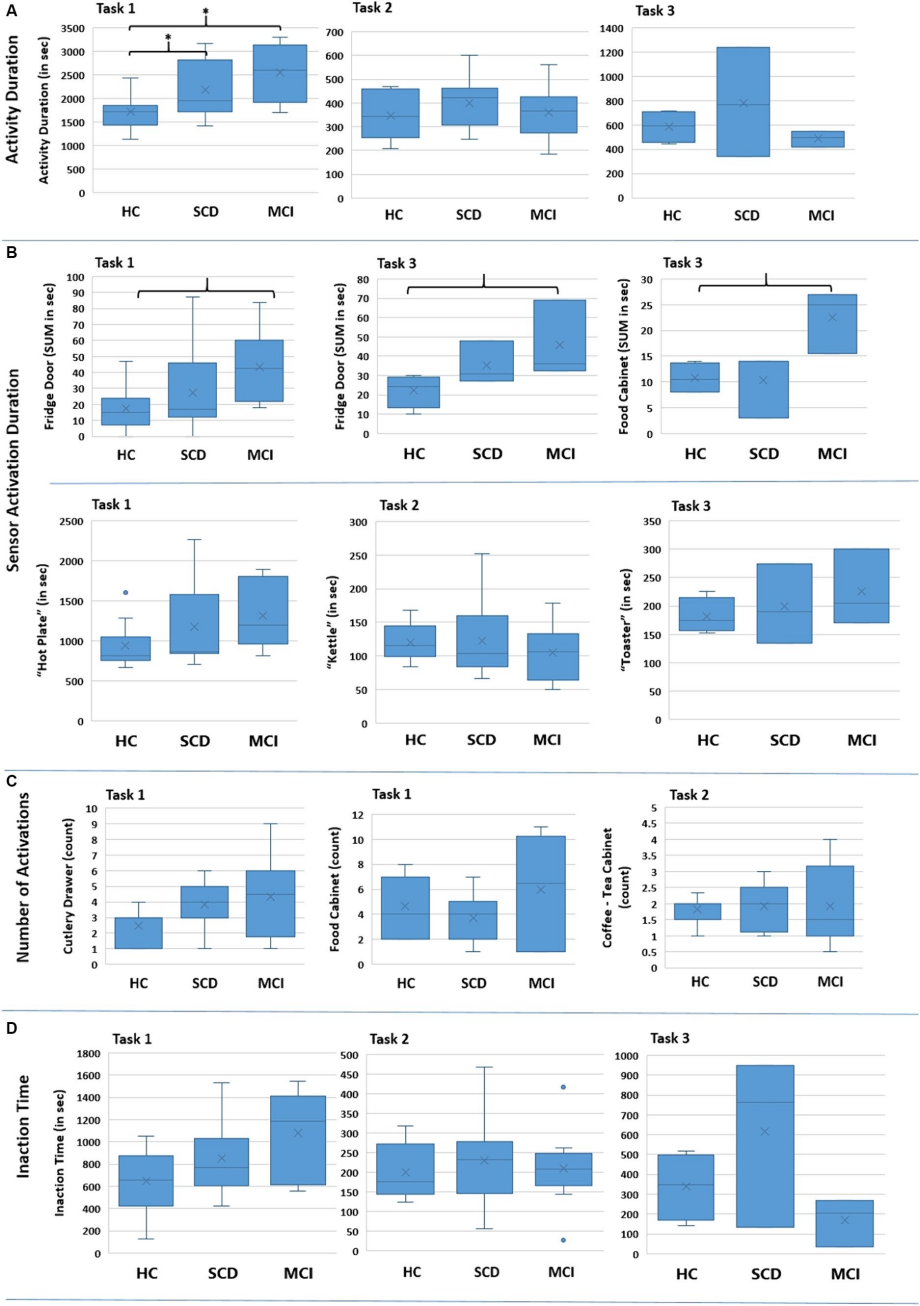
Boxplots showing the distribution of the collected data for the various features for the three groups regarding each Task (Task 1 – Meal Preparation, Task 2 – Beverage Preparation, Task 3 – Snack Preparation). The * in **(A)** indicates the group pairs where a statistical significance at *p* = 0.05 level was found. The brackets in **(B)** indicate the group pairs where a weak trend (*p* = 0.057) was found. No statistical significance was found in **(C)** and **(D)** between groups.

Furthermore, the time needed to complete a Task was assessed. The distribution of the collected data is presented in Figure 7A for the three Tasks. The results of the Kruskal-Wallis test for the “Activity Duration” feature for the three Tasks showed a statistically significant difference across the three groups of participants at a *p* = 0.05 level, in Task 1 – Meal Preparation [H (2) = 7.607, *p* = 0.022] (Table 5). No statistically significant difference was noted for “Activity Duration” in Tasks 2 and 3.

**Table 5:** Descriptive statistics (mean value and standard deviation, given in seconds and count according to each feature), Kruskal–Wallis and Mann–Whitney *p* values for the explored features regarding the performed Tasks (Task 1 – Meal Preparation, Task 2 – Beverage Preparation, Task 3 – Snack Preparation).

Task	Sensor	HC	SCD	MCI	Kruskal Wallis *p*-value	Mann–Whitney U-test *p*-value			Mean value (standard deviation)				HC versus SCD	HC versus MCI	SCD versus MCI
*Feature “Activity Duration” (in seconds)*								
1	All sensors comprising the corresponding ADL are taken into account	1710 (349)	2,180 (604)	2,546 (619)	**0.022**	**0.040**	**0.015**	0.350
2		346 100	400 (104)	362 (106)	0.566	–	–	–
3		586 (135)	780 (450)	487 (66)	0.546	–	–	–
*Feature “Sensor Activation Duration” (in seconds)*								
1	Fridge door	17 (14)	27 (26)	44 (24)	0.074	**–**	–	–
3	Fridge door	22 (9)	35 (11)	46 (20)	**0.046**	–	0.057	–
3	Food cabinet	11 (3)	9 (8)	23 (6)	**0.050**	–	0.057	–
1	Hot plate	941 (282)	1,176 (546)	1,314 (444)	0.139	–	–	–
2	Kettle	120 (29)	123 (53)	117 (56)	0.963	–	–	–
3	Toaster	182 (31)	200 (70)	226 (67)	0.554	–	–	–
*Feature “Number of Activations” (count)*								
1	Cutlery drawer	2.45 (1.13)	3.82 (1.60)	4.33 (2.80)	0.093	–	–	–
1	Food cabinet	4.64 (2.46)	3.73 (1.95)	6.00 (4.38)	0.517	–	–	–
2	Coffee – Tea cabinet	1.81 (0.13)	1.92 (0.21)	2.03 (0.38)	0.984	–	–	–
*Feature “Inaction Time” (in seconds)*								
1	Time period during a Task where no sensors were activated	647 (284)	854 (305)	1,081 (401)	0.148	–	–	–
2		200 (70)	230 (111)	210 (103)	0.762	–	–	–
3		339 (171)	616 (428)	169 (120)	0.394	**–**	**–**	**–**

Bold values denote statistical significance at the *p* < 0.05 level.

Afterwards, in order to determine the groups between which discrimination was possible in Task 1 – Meal Preparation, Mann Whitney test was performed, showing that the duration was statistically significant longer for the SCD group compared to HC (U = 29.00, *p* = 0.040), and also for the MCI group compared to HC (U = 9.00, *p* = 0.015). No differentiation was possible between the SCD and the MCI group for Task 1.

#### 3.1.4 Individual sensors

As each Task consists of a synthesis/composition of Events, signalled by different sensors, it was considered important to investigate next the activation duration as well as the number of activations marked for the individual sensors. It is noted that six sensors were placed to monitor Task 1 – Meal Preparation, five for Task 2 – Beverage Preparation, and six for Task 3 – Snack Preparation (Table 4 in previous section).

##### 3.1.4.1 Sensor activation duration

Kruskal Wallis test was performed for all available sensors regarding the features “sum_ < name_of_sensor>” and “avg_ < name_of sensor> (Supplementary information).

A statistically significant difference was found only for the duration of the sensors placed on the Fridge Door and the Food Cabinet in Task 3 – Snack Preparation, while a weak trend was observed for the Fridge Door sensor in Task 1 – Meal Preparation (Table 5). Mann Whitney tests for the sensors of Task 3, revealed a trend between the HC and MCI groups, showing that the MCI group noted longer durations when utilizing the Fridge Door and the Food Cabinet during the Snack Preparation task. The data distribution of the abovementioned sensors can be found in Figure 7B.

Regarding the use of the small electrical appliances, no differentiation was possible. Boxplots showing the distribution among groups are presented in Figure 7B, while descriptive statistics and the results from the statistical tests are also included in Table 5.

##### 3.1.4.2 Number of activations (count per sensor)

No interesting finding could be noted here. Even though this feature could be connected with the performed number of steps, where for Task 1 – Meal Preparation the MCI exhibited a larger number of mean steps, no statistically significant difference could be found. Indicative examples of the data obtained can be seen in Figure 7C and Table 5.

#### 3.1.5 Inaction time

The composite feature “Inaction Time,” aiming to capture the time participants spent during an activity without activating a sensor (e.g., due to wandering, considering their next action), did not yield any differences between groups. Considerable overlapping between groups is noted for “Inaction Time,” and no differences emerged from the Kruskal-Wallis tests performed (Figure 7D; Table 5).

### 3.2 Sensitivity and specificity

The potential utility of the three ADL tasks as objective markers to distinguish an individual’s cognitive condition (SCD, MCI) compared to HC by testing Sensitivity and Specificity among the groups (Table 6) was investigated.

**Table 6:** Sensitivity and specificity of the three ADL tasks (Task 1, Task 2 and Task 3) regarding the feature “Activity Duration” for discriminating between groups.

Feature “activity duration”		AUC	Threshold value (in seconds)	Sensitivity	Specificity
HC versus SCD	Task 1 – Meal preparation	**0.76**	1715	0.82	0.64
	Task 2 – Beverage preparation	0.62	342	0.67	0.56
	Task 3 – Snack preparation	0.67	700	0.67	0.75
HC versus MCI	Task 1 – Meal preparation	**0.86**	1924	0.83	0.82
	Task 2 – Beverage preparation	0.58	352	0.70	0.56
	Task 3 – Snack preparation	**0.75**	500	0.75	0.67
SCD versus MCI	Task 1 – Meal preparation	0.65	1977	0.83	0.55
	Task 2 – Beverage preparation	0.59	403	0.58	0.80
	Task 3 – Snack preparation	0.67	522	0.67	0.67

The sensitivity and specificity scores corresponding to the cut-off thresholds alongside with the AUC. Bold values suggest acceptable discrimination (0.7–0.8) or excellent discrimination (0.8–0.9), (Hosmer and Lemeshow, 2000; Mandrekar, 2010).

In general, an AUC of 0.5 suggests no discrimination (i.e., ability to diagnose patients with and without the disease or condition based on the proposed test), 0.7 to 0.8 is considered acceptable, 0.8 to 0.9 is considered excellent, and more than 0.9 is considered outstanding (Hosmer and Lemeshow, 2000; Mandrekar, 2010).

In detail, we managed to successfully discriminate HC from SCD in Task 1 – Meal Preparation (AUC = 76%, Sensitivity = 0.82 and Specificity = 0.64) regarding the “Activity Duration” feature. Also, we managed to discriminate HC from MCI in Task 1 – Meal Preparation (AUC = 86%, Sensitivity = 0.83 and Specificity = 0.82) and Task 3 – Snack Preparation (AUC = 75%, Sensitivity = 0.75 and Specificity = 0.67). Interestingly, no discrimination could be made between the SCD and MCI groups. The “Activity Duration” feature of the Meal Preparation Task can distinguish between HC-MCI and HC-SCD with acceptable robustness.

### 3.3 Usability and satisfaction questionnaires

The overall experience as perceived by the participants during the study in the SH was assessed with a study satisfaction questionnaire that referred to the visit, the tasks, the time needed to complete the tasks and the level of difficulty (Supplementary information). No difference between the three groups could be noted regarding the given feedback. The majority of the participants (72%) when asked if they were satisfied with their participation in the study, replied with “Extremely satisfied.” The participants perceived the study as “Extremely appealing” (60%), “Very appealing” (22%) and “Appealing” (14%). The time planned for the Tasks was found to be sufficient with participants commenting that they did not need more time to complete the activities (94%). The instructions and task descriptions were unanimously found to be extremely easy to read and understand, and the labels placed to mark the different cabinets (labelled “Food,” “Cutlery”) were perceived as very useful. No technical issues and no issues of any other nature were noted during the participants’ visit (e.g., problems with the sensors, person wanting to terminate participation). All participants replied with “No” when asked if any skills were required to interact with the proposed technologies. Describing the overall visit, all participants replied that their participation was a positive experience and no feeling of inconvenience was noted (e.g., stress, depression, anxiety). Additionally, the mean scores (M) per group, for the SUS questionnaire (scores can range from 0 to 100) (Brooke, 1986), revealed excellent overall usability [HC: M = 94 (SD = 5.8), SCD: M = 92.9 (SD = 4.7), MCI: M = 93.9 (SD = 5.2)]. The PANAS questionnaire, designed to measure emotional experience (namely positive affect, PA and negative affect, NA was utilized; Watson et al., 1988). Respondents are asked to indicate the extent to which they have experienced each emotion (e.g., excitement, sadness) over a specific period of time rating them on a scale from 1 to 5 (PA and NA can range from 10 to 50). The participants showed acceptable positive and negative affects, while no differences between groups were observed [HC: M_PA_ = 36 (5), M_NA_ = 20 (5), SCD: M_PA_ = 35 (3), M_NA_ = 19 (6), MCI: M_PA_ = 36 (5), M_NA_ = 21 (6)].

## 4 Discussion

The Smart Home, simulating a domestic residence, offers a unique environment allowing for controllable experimental conditions. Through data collection using non-intrusive fixed in-home sensors in the

CERTH-ITI SH, and instructing participants to follow a protocol listing a number of ADLs, we aimed to capture, quantify and assess ADL performances, as these can lead to insightful measures for functional deterioration. Through this ecologically valid assessment, we aimed to detect changes between three different cohorts, namely HC, participants with SCD and participants with MCI. Visualization of the collected data and extraction of meaningful features in the form of a dataset available for analysis was possible by utilizing the CARL platform.

This preliminary investigation demonstrated that SH technologies present an opportunity for an unbiased and real-world evaluation of ADLs in individuals with SCD and MCI. The study allowed for the assessment of not only whether a task is accomplished but also how it is carried out.

Discussing protocol adherence and number of overall completed ADLs, it appeared that participants did not follow precisely the provided protocol with the step-by-step task descriptions, but proceeded with the ADLs in a more intuitive way. Additionally, commenting on the number of steps needed to complete a Task, only for Task 1 – Meal Preparation was a small difference observed in the mean number of steps for the MCI group compared to the HC and SCD groups. In Tasks 2 – beverage preparation and 3 – snack preparation the three groups performed similar number of steps.

The correctness of the executed steps may not be easily assessed, using simple statistical analysis methods, as the step sequence differs not only between groups but also notably, within groups as well. However, as participants proceeded with the Task execution in a freely manner, the observations made are in the context of real-environment monitoring and allow real-life evaluations. Additionally, as commented in Jekel et al. (2016), we should consider that there could be significant individual variability in performing a task in a correct manner, for this, it can be overall argued, if correctness of steps can pose a useful feature. Also, in the work of Lundström et al. (2016) guidelines provided to participants for performing tasks (e.g., prepare breakfast, get hot drink, prepare dinner), were written in a simplified manner to allow for natural variation.

It is noted that overall the HC and SCD groups performed, respectively, 70 and 74% of the expected tasks and the MCI group 58%. Specifically for Task 1 – Meal Preparation, the MCI group exhibited the lowest number of performance compared to the other groups (11/12 HC, 12/13 SCD, 7/11 MCI). No plausible justification could be derived for this discrepancy.

Regarding activity duration, Task 1 – Meal Preparation, yielded differences between the groups, which constitutes an interesting finding. It was considered that the more elaborate task of preparing a hot meal, due to its added complexity, was able to highlight the groups’ differences attributed to functional decline due to cognitive impairment. Specifically, comparison of participants’ performances in Task 1 led to statistically significant differences between groups, namely between HC versus SCD, and HC versus MCI, based on the time needed to complete the task. It is noteworthy that no differentiation could be made between SCD and MCI participants.

The meal preparation task has been investigated also in a different context, in the work of Atkins et al. (2015, 2018), where the Virtual Reality Functional Capacity Assessment Tool (VRFCAT) was used. Discriminating healthy older adults from older adults with SCD was possible, as the latter noted a statistically significant larger amount of time to complete the given tasks and performed more errors.

Furthermore, ROC Curve values were encouraging for the Task 1- Meal Preparation, reaching 86% in the classification of HC vs. MCI and exciding 70% in the classification of HC vs. SCD. This is a promising finding, as available neuropsychological tests do not discriminate SCD from HC (Sikkes et al., 2009; Kaur et al., 2016).

On the other hand, the more straightforward / simple tasks of preparing a beverage (Task 2), and a snack (Task 3) were not able to show between groups differences. This is in accordance with existing literature. In Jekel et al. (2016), the coffee and sandwich preparation tasks were also not able to differentiate the HC and MCI participants, while in Karakostas et al. (2020) assessing various ADLs, no difference could be observed between HC and MCI for the tea preparation task. As has been commented in Jekel et al. (2016), these tasks could be considered as not highly cognitive demanding.

Additionally, regarding the individual sensors, only the ones placed on the Fridge Door and the Food Cabinet (both Fridge and Food Cabinet entailing a variety of different products) could show some difference between groups in their utilization (weak trends). Again, we are of the opinion that the fridge and the cabinet containing a number of products could be considered as the more complicated to handle.

The feature “Inaction Time” was considered promising as it was assumed that cognitive impairment and functional decline could lead to increased wandering time between actions due to possible disorientation (Coughlan et al., 2018). While the participants’ data distribution for “Inaction Time” in Task 1 – Meal Preparation showed this expected tendency, no statistical significant difference was observed.

The duration of utilizing the small electrical appliances was compared between groups. Since the activation of these appliances was seen as a requirement for the formation of the ADLs (the ADLs were built around the data collected from the small electrical appliances), it was important to determine if this factor predominantly influenced the overall composite ADL duration feature. However, no statistically significant difference could be observed between groups.

In general, for many of the collected sensor data, descriptive statistics revealed an initial trend that MCI participants (and in some cases SCD participants) exhibit longer durations than HC, but significant overlapping exists between the groups not allowing further comparisons. Regarding the features addressing aspects besides duration, like the number of steps needed to complete a Task, the number of repetitions in utilizing, e.g., specific cupboards, could not be used to differentiate the groups. We are of the opinion that these features are reflecting actions not cognitive demanding and are not granular enough to highlight differences. For this, further feature exploration is needed to gain additional markers from the performed ADLs.

The present study shows that implementing new technologies that are able to detect subtle changes in cognitive and functional patterns may allow earlier diagnosis, even at the point when memory functions are still intact, such as the SCD stage.

While studies on activity recognition from collected sensor data are available in the literature (Bouchabou et al., 2021), there is limited research on efforts of quantifying and comparing the performed ADLs among early stages of cognitive impairment (Atkins et al., 2018), while only a comparison between a small number of HC and MCI participants has been attempted so far (Stucki et al., 2014; Jekel et al., 2016; Stavropoulos et al., 2016; Urwyler et al., 2017; Karakostas et al., 2020).

Moreover, there is scarce evidence for real-life, smart home-based use of technologies for early detection of dementia, and no approach is yet perceived as mature enough (Piau et al., 2019). An exception can be considered the Collaborative Aging Research Using Technology (CART) Initiative, a multi-site, nationwide project (Thomas et al., 2021; Bernstein et al., 2022). The study uses multiple embedded sensing technology and diverse data to support research in the field of health and independent living, focusing on older adults from various communities. However, as the authors note, further proof is needed on the precision, accuracy and reliability of these novel outcome measures before home-based sensor technologies can be included in clinical trials and utilized in the monitoring of chronic diseases (Thomas et al., 2021).

A frequent constraint in the majority of studies that evaluate SH technologies for monitoring ADLs, is their lack of focus on participants’ acceptance of the devices, as indicated by a recent systematic review (Lawson et al., 2023). Along with the fact that elderly participants are not very keen on using smart technologies (Tiersen et al., 2021; Wei et al., 2023), participants views need to be considered when introducing new technologies. The present study and the proposed technologies were evaluated by the participants, and were regarded as feasible. Participants answered in a positive manner when asked a number of questions regarding their experience and their stay, the sensors and technologies utilized, while they did not experience any issues.

The study has some limitations that need to be acknowledged. While the sample size (N = 36) could be considered sufficient, considering the exploratory nature of the study and existing literature (Hayes et al., 2008; Petersen et al., 2015; Seelye et al., 2020) it is noted that, as some participants did not complete all tasks listed in the protocol, the dataset was further decreased. For example, a number of people, independently of their group, did not perform the Task 3-Snack Preparation activity (7/12 HC, 9/13 SCD and 8/11 MCI). This could be attributed to the fact that as participants stated, “They were not hungry,” or “preferred to rest some more” and “explore the Smart Home’s premises instead.” This led to a restricted dataset available for Task 3 for analysis, the findings of which must be viewed with caution.

Additionally, it is noted that, as participants visited a new, unknown to them environment, this could also have affected the way they performed the various ADLs. Nevertheless, effort was made to simulate a real domestic environment while also adequate time was provided to the participants to feel comfortable in the house and discuss any concern with the researchers.

During feature extraction, conversion of raw data to events and activities involved refinement through post-processing. Even though all data processing was performed in a systematic manner and is described in the text, and a validation of the ADLs derived from the sensors was performed using collected information as ground truth, in the interest of thoroughness this is acknowledged as a potential limitation.

Also, the use of flood sensors was investigated, installed in the kitchen (sink) and the bathroom (sink and flush). However, as the sensors are designed to detect water leaks and flooding, the necessary adjustments made to the sensors to monitor water usage instead, did not allow robust and continuous data collection. For this, the sensors were not included in the study.

Furthermore, it is noted that, as biofluid biomarkers were not collected for all participants, the etiology of the MCI and SCD group cannot be distinguished (amyloid positive vs. amyloid negative).

A limitation of the study, to be addressed in future work, constitutes the absence of a comparison/correlation to relevant conventional measures of function [e.g., the informant-based Amsterdam IADL questionnaire (Sikkes et al., 2012), the Naturalistic Action Test (Seligman et al., 2014)].

Finally, regarding the study’s feasibility assessment, as researchers were present while participants filled out the questionnaires, possible bias could occur.

The herein presented SH study provides a proof-of-concept for the feasibility of identifying, quantifying and assessing ADLs and differentiating known-groups via monitoring their performance. It is evident that new tools will be required to assess and evaluate clinically significant changes (Atkins et al., 2015; Gold et al., 2018). The inclusion of people in preclinical stages of AD, constitutes an important step towards the advancement of digital biomarkers.

## 5 Conclusion

Participants spent a day in CERTH-ITI’s Smart Home, a controlled environment that simulates a fully functional house, and were asked to perform a number of ADLs according to a given protocol. The results proved the differentiation among the HC group compared to the SCD and the MCI groups considering the feature “Activity Duration” in Task 1 - Meal Preparation. Task 1 can be considered more complex compared to Task 2 - Beverage Preparation and Task 3 – Snack Preparation.

The distinction of the SCD from the HC group, constitutes an important finding, as conventional assessments (neuropsychological questionnaires) note no difference between these groups. Furthermore, the differentiation of HC and MCI participants, as documented in the existing literature, confirms the study design and the methodology followed. Additionally, it is interesting to note that no significant group differences could be observed between the SCD and the MCI groups.

These findings further support the interest and need to include people in preclinical stages of dementia in current research. Furthermore, the study was proven feasible, with participants expressing positive feedback for the study and the technologies used.

Access to this information, paves the way for detection of behavioural patterns and deviations allowing for early observation of deterioration in function. This ecologically valid study provides evidence that ADL performance can be utilized and further evolved to develop clinically relevant digital biomarkers. These biomarkers could serve for monitoring participants in at-home settings, participant stratification as well as secondary endpoints in clinical trials to complement established outcome measures.

Starting from these encouraging findings, further research would be needed to determine the long-term reliability and predictive value of the proposed assessment tools in the clinical practice. Consecutive data collection on the executed ADLs over an extended period of time, would allow us to monitor behavioral patterns of the individuals in depth, identify personalized thresholds and highlight potential functional deterioration. Additionally, in this way, other factors could be controlled for and tested. For example, measures of sleep duration and quality could be incorporated (by using wearable devices, or pressure sensors placed underneath the mattress on the bed) to better understand their influence on ADL performance. A longitudinal study could evaluate and strengthen the presented findings and provide a useful tool, to serve as a secondary endpoint in drug trials on the therapeutic efficacy of prescribed drugs.

Furthermore, while study centers are not widely available for healthcare research, we envision that as technology continues to evolve and becomes increasingly part of our everyday life, the suggested assessment could be implemented in home environments, facilitating the inclusion of people in rural areas. In detail, the integration of smart devices and appliances, outfitted with microprocessors and WiFi access, is steadily gaining prominence within domestic settings. This reflects a significant shift towards the adoption of interconnected technologies in everyday life. The proposed approach is scalable and cost-effective. The protocol deploys commercially available sensors, indicating its practicality and accessibility. Additionally, the developed CARL platform is device agnostic, allowing the integration of different sensors and demonstrating flexibility in technological advancements.

As a first step towards the implementation and exploration of testing this protocol at a home environment, another sub-study realized in the context of the RADAR-AD project was set up to explore the feasibility of such an approach. The fixed in-home sensors were placed in participants’ homes and data collection was ongoing for 4 weeks.

## Data availability statement

The datasets generated and/or analysed during the current study are available from the corresponding author on reasonable request.

## Ethics statement

The studies involving humans were approved by the Ethics Committee of the Centre of Research and Technology Hellas (CERTH) and the Scientific and Ethics Committee of the Greek Association of Alzheimer’s Disease and Related Disorders (GAADRD). The studies were conducted in accordance with the local legislation and institutional requirements. The participants provided their written informed consent to participate in this study.

## Group members of RADAR-AD

Dag Aarsland, Halil Agin, Vasilis Alepopoulos, Alankar Atreya, Sudipta Bhattacharya, Virginie Biou, Joris Borgdorff, Anna-Katharine Brem, Neva Coello, Pauline Conde, Nick Cummins, Jelena Curcic, Casper de Boer, Yoanna de Geus, Paul de Vries, Ana Diaz, Richard Dobson, Aidan Doherty, Andre Durudas, Gul Erdemli, Amos Folarin, Suzanne Foy, Holger Froehlich, Jean Georges, Dianne Gove, Margarita Grammatikopoulou, Kristin Hannesdottir, Robbert Harms, Mohammad Hattab, Keyvan Hedayati, Chris Hinds, Adam Huffman, Dzmitry Kaliukhovich, Irene Kanter-Schlifke, Ioannis Kompatsiaris, Ivan Koychev, Rouba Kozak, Julia Kurps, Sajini Kuruppu, Claire Lancaster, Robert Latzman, Ioulietta Lazarou, Manuel Lentzen, Federica Lucivero, Florencia Lulita, Nivethika Mahasivam, Nikolay Manyakov, Emilio Merlo Pich, Peyman Mohtashami, Marijn Muurling, Vaibhav Narayan, Vera Nies, Spiros Nikolopoulos, Andrew Owens, Marjon Pasmooij, Dorota Religa, Gaetano Scebba, Emilia Schwertner, Rohini Sen, Niraj Shanbhag, Laura Smith, Meemansa Sood, Thanos Stavropoulos, Pieter Stolk, Ioannis Tarnanas, Srinivasan Vairavan, Nick van Damme, Natasja van Velthogen, Herman Verheij, Pieter Jelle Visser, Bert Wagner, Gayle Wittenberg, and Yuhao Wu.

## Author contributions

MG: Data curation, Formal analysis, Investigation, Writing – original draft, Writing – review & editing, Validation, Visualization. IL: Writing – original draft, Writing – review & editing, Conceptualization, Formal analysis, Investigation. VA: Data curation, Investigation, Software, Validation, Visualization, Writing – original draft, Writing – review & editing, Formal analysis. LM: Writing – review & editing, Investigation. VO: Writing – review & editing, Investigation. TS: Conceptualization, Funding acquisition, Supervision, Writing – review & editing, Methodology. SN: Conceptualization, Funding acquisition, Supervision, Writing – review & editing, Methodology, Project administration. IK: Conceptualization, Funding acquisition, Supervision, Writing – review & editing, Methodology, Project administration. MT: Investigation, Supervision, Writing – review & editing.

## Footnotes

^1^ https://www.radar-ad.org/

^2^FIBARO sensors: https://www.fibaro.com/en/products/all-domotica-devices/

^3^ https://smarthome.iti.gr/

^4^ http://www.alzheimer-hellas.gr/index.php/el/

^5^ https://www.radar-ad.org/sites/radarad/files/2021-02/RADAR-AD%20device%20selection%20report.pdf

^6^ https://www.alzheimer-europe.org/about-us/european-working-group-people-dementia

^7^ https://www.alzheimer-europe.org/

^8^ https://www.fibaro.com/en/products/home-center-lite/
