# Supplementary material for: Assessing the cognitive decline of people in the spectrum of AD by monitoring their activities of daily living in an IoT-enabled smart home environment: a cross-sectional pilot study
Source: Front Aging Neurosci. 2024 Mar 28;16:1375131. doi: 10.3389/fnagi.2024.1375131 (PMC11007144; doi:10.3389/fnagi.2024.1375131)
Supplement: Supplementary file 1 [file Data_Sheet_1.PDF]

Supplementary Information

1.1 Study Protocol

A. One-day Visit

Day 1
09:00-10:00 Reception, acquaintance with the Smart Home and wearables
10:00-11:00 Hot beverage preparation (**Task A**) / time for TV - reading and adapting
11:00-13:00 Yoga & Mindfulness (**Task B**)
13:00-15:00 Preparing a hot meal (**Task C**)
15:00-16:00 Time for TV / reading / lunch
16:00-17:00 Hot beverage preparation (**Task A**) and snack preparation (**Task D**)
17:00-18:00 Completion of a questionnaire and departure of participant

B. Visit with overnight stay

Day 1
09:00-10:00 Reception, acquaintance with the Smart Home and wearables
10:00-11:00 Hot beverage preparation (**Task A**) / time for TV - reading and adapting
11:00-13:00 Yoga & Mindfulness (**Task B**)
13:00-14:00 Time to rest / take a bath*
13:00-15:00 Preparing a hot meal (**Task C**)
15:00-16:00 Time for TV / reading / lunch
16:00-17:00 Hot beverage preparation (**Task A**)
17:00-18:00 Completion of questionnaire
18:00-20:00 Snack preparation (**Task D**)
22:00 (indicative) Individual Relaxation Exercises and Sleep (**Task E**)
*Available bathroom for the participants to wash/bath

Day 2
09:00-10:00 Beverage Preparation (**Task A**)
10:00-11:00 Snack Preparation (**Task D**)
11:00-12:00 Completion of questionnaires and departure of participant

The given times are indicative - participants can start the activities as they please

----

1.2 Step by step Task Descriptions

Task 1 – Meal Preparation

Please follow the instructions below to prepare a hot meal

- Open the cabinet labelled “Dishes and Cups” and take the large pot and the measuring cup
- Open the cabinet labelled "Food" and take out the barley, the tomato juice, salt, pepper, olive oil and oregano
- Open the drawer labelled “Cutlery” and take out a spoon
- Use the measuring cup to add to the pot the following:
    - 2 and a half measuring cups of water
    - 1 measuring cup tomato juice
    - half a cup of olive oil
    - 1 tbsp. oregano (optional)
    - Add salt to taste
    - Add pepper to taste
    - Mix the ingredients with the spoon
- Place the pot with the ingredients on the electric stove and turn it on
- As soon as the food starts to boil, lower the temperature and add 1 measuring cup of barley
- Stir for another 2 minutes and bring again to boil for another 5 minutes
- Turn off the electric stove and leave the pot covered with the lid for 15 minutes
- Open the fridge and take out the package with the feta cheese
- Cut a piece and add it into the pot
- Open the cabinet labelled “Food” and return the ingredients used
- Once the 15 minutes are up your meal is ready
- Open the cabinet labelled “Dishes and Cups” and take out a plate and serve your meal
- Bon appetite!

Task 2 – Beverage Preparation

Please follow the instructions below to prepare a hot beverage

- Open the cabinet labelled “Coffee - Tea”
- Take out the container with the instant coffee or tea
- Take out the container with the sugar (optional only if you put sugar in your beverage)
- Take a cup from the cabinet labelled “Dishes and Cups”
- Open the drawer labelled “Cutlery” and take a teaspoon
- Add the corresponding amount of coffee or tea in the cup
- Open the tap in the sink and place an amount of water in the electric kettle
- Turn the kettle on to heat up the water
- Once the water is heated turn off the electric kettle
- Add the hot water from the kettle to the cup
- Turn off the kettle once the beverage is ready
- Do not unplug the electric kettle
- Make sure you have turned the kettle off
- Enjoy your beverage!

- After drinking your beverage, wash the cup and the kettle with the dish sponge and leave them in the sink to dry

Task 3 – Snack Preparation

Please follow the instructions below to prepare a snack

- Open the cabinet labelled "Food" and get the bread
- Take a plate from the cabinet labelled "Dishes and Cups"
- Open the drawer labelled "Cutlery" and take out a fork and a knife
- Open the refrigerator and get the sliced cheese and turkey (optional)
- Place cheese and turkey between 2 slices of bread
- Turn on the toaster by plugging it into the socket
- Place your sandwich on the toaster
- Place the cheese and turkey back in the refrigerator
- As soon as the toaster light goes out, remove it from the socket
- Place your grilled sandwich on your plate
- Make sure you have turned the toaster off

1.3 Results

1.3.1 Exploring ADLs – Task Comparison between groups

Descriptive statistics and results for the statistical tests are given for all features. It is noted that herein all the results are presented, while in the manuscript only the more prominent for discussion features are given in Figures and Tables to provide the reader with a clearer overview.

Table 1 Mean (and Standard Deviation) for the individual features of the three ADL activities (Task 1 – Meal Preparation, Task 2 – Beverage Preparation and Task 3- Snack Preparation) across the three groups. P-values for comparisons between the three groups are derived from Kruskal Wallis tests, while the three last columns reflect Mann-Whitney U-tests showing between group comparisons.

			HC	SCD	MCI	Kruskal-Wallis	Mann Whitney U test p-value		
**ADL Tasks**	**Available data**	**Feature**	**M (SD)**			**p-value**	**HC vs SCD**	**HC vs MCI**	**SCD vs MCI**
**Task 1- Meal Preparation**	11/12 HC, 11/13 SCD, 6/11 MCI	**Activity Duration Task 1**	1710 (349)	2180 (604)	2546 (619)	**0.022**	**0.040**	**0.015**	0.350
		**sum_Dishes Cabinet**	13 (10)	16 (15)	22 (25)	0.954	-	-	-
		**avg_Dishes Cabinet**	4.3 (2.3)	6.2 (4.6)	6.3 (4.6)	0.616	-	-	-
		**count_Dishes Cabinet**	2.5 (1.8)	1.9 (1.3)	2.3 (2.3)	0.788	-	-	-
		**sum_Hot plate**	941	1176	1314	0.139	-	-	-

			(282)	(546)	(444)				
		**sum_Food Cabinet**	62 (44)	65 (44)	51 (27)	0.908	-	-	-
		**avg_Food Cabinet**	15 (9)	22 (18)	12 (7)	0.519	-	-	-
		**count_Food Cabinet**	4.6 (2.4)	3.7 (1.9)	6 (4.3)	0.517	-	-	-
		**sum_Trash Cabinet**	16 (15)	19 (9)	16 (19)	0.529	-	-	-
		**avg_Trash Cabinet**	5.2 (2.8)	8 (2.8)	6.9 (3.1)	0.169	-	-	-
		**count_Trash Cabinet**	2.6 (2)	2.5 (0.9)	2.5 (1.8)	0.529	-	-	-
		**sum_Cutlery Drawer**	15 (7)	22 (8)	18 (8)	0.135	-	-	-
		**avg_Cutlery Drawer**	5.7 (1.4)	5.5 (1.4)	6.3 (2)	0.753	-	-	-
		**count_Cutlery Drawer**	2.4 (1.1)	3.8 (1.6)	4.3 (2.8)	0.093	-	-	-
		**sum_Fridge Door**	17 (14)	27 (26)	44 (24)	0.074	-	-	-
		**avg_Fridge Door**	7.9 (5.4)	8 (5.2)	20 (12)	0.085	-	-	-
		**count_Fridge Door**	1.8 (1.2)	2.2 (2.6)	3 (2.7)	0.666	-	-	-
		**sum_Inaction Time**	647 (284)	854 (305)	1081 (401)	0.148	-		-
**Task 2- Beverage Preparation**	9/12 HC, 12/13 SCD, 10/11 MCI	**Activity Duration Task 2**	346 100	400 (104)	362 (106)	0.566	-	-	-
		**sum_Kettle**	120 (29)	123 (53)	117 (56)	0.963	-	-	-
		**sum_Dishes Cabinet**	7 (8)	10 (6)	20 (26)	0.499	-	-	-
		**avg_Dishes Cabinet**	4 (3.5)	6.7 (3)	13 (21)	0.264	-	-	-
		**count_Dishes**	1.1 (0.8)	1.33 (0.7)	0.8 (0.9)	0.327	-	-	-
		**sum_Coffee - Tea Cabinet**	17 (10)	23 (12)	26 (19)	0.251	-	-	-
		**avg_Coffee-Tea Cabinet**	12 (9)	17 (13)	16 (13)	0.669	-	-	-
		**count_Coffee-Tea Cabinet**	1.8 (0.4)	1.9 (0.7)	1.9 (1.2)	0.984	-	-	-
		**sum_Trash Cabinet**	6 (5)	8 (6)	4 (5)	0.224	-	-	-
		**avg_Trash Cabinet**	4 (2.5)	5 (2.7)	2.3 (3)	0.144	-	-	-
		**count_Trash Cabinet**	0.7 (0.6)	1.3 (1)	0.4 (0.6)	0.081	-	-	-
		**sum_Cutlery Drawer**	6 (3)	6 (3)	8 (6)	0.406	-	-	-
		**avg_Cutlery Drawer**	3.8 (2)	4.2 (2)	4.5 (2.5)	0.879	-	-	-
		**count_Cutlery Drawer**	1.2 (0.6)	1.2 (0.6)	1.3 (0.7)	0.468	-	-	-
		**sum_Inaction Time**	200 (70)	230 (111)	210 (103)	0.762	-	-	-

**Task 3- Snack Preparation**	4/12 HC, 3/13 SCD and 3/11 MCI	**Activity Duration Task 3**	586 (135)	780 (450)	487 (66)	0.546	-	-	-
		**sum_Toaster**	182 (31)	200 (70)	226 (67)	0.554	-	-	-
		**sum_Dishes Cabinet**	6 (5)	7 (7)	12 (4)	0.249	-	-	-
		**avg_Dishes Cabinet**	4.1 (2.8)	5.1 (4.6)	7.8 (2)	0.249	-	-	-
		**count_Dishes Cabinet**	0.9 (0.9)	1 (1)	1.1 (0.3)	0.807	-	-	-
		**sum_Food Cabinet**	11 (3)	9 (8)	23 (6)	**0.050**	-	0.057	-
		**avg_Food Cabinet**	8 (5.7)	5 (4.5)	13 (4.5)	0.050	-	-	-
		**sum_Trash Cabinet**	8 (5)	4 (3)	5 (4)	0.284	-	-	-
		**avg_Trash Cabinet**	7.6 (4.5)	3 (3)	4.8 (4.2)	0.286	-	-	-
		**count_Trash Cabinet**	0.9 (0.3)	0.7 (0.6)	0.5 (0.5)	0.519	-	-	-
		**sum_Cutlery Drawer**	7 (4)	7 (10)	11 (1)	0.544	-	-	-
		**avg_Cutlery Drawer**	4.5 (1.5)	3 (3)	7 (2.7)	0.328	-	-	-
		**count_Cutlery Drawer**	1.5 (1)	1.3 (1.5)	1.7 (0.6)	0.803	-	-	-
		**sum_Fridge Door**	22 (9)	35 (11)	46 (20)	**0.046**	-	0.057	-
		**avg_Fridge Door**	7.8 (2)	8.5 (2)	19 (3.5)	**0.050**	-	0.057	-
		**count_Fridge Door**	2.5 (1)	4 (1)	2.3 (0.6)	0.103	-	-	-
		**sum_Inaction Time**	339 (171)	616 (428)	169 (120)	0.394	-	-	-

1.3.2 Usability and Satisfaction Questionnaire

1.3.2.1 Study Satisfaction Questionnaire

1. Overall, how satisfied were you participating in the Tier 3 Smart Home Study?
   a. Extremely well
   b. Very well
   c. Somewhat well
   d. Not so well
   e. Not at all well

2. How appealing did you find the Study?
   a. Extremely appealing
   b. Very appealing
   c. Somewhat appealing
   d. Not so appealing
   e. Not at all appealing

3. Did it take you more or less time than expected to complete the tasks?
   a. A lot less time
   b. A little less time
   c. About what I expected
   d. A little more time
   e. A lot more time

4. Did you need more time to complete the tasks?
   a. Yes b. No

5. Would you like to increase the time of each task?
   a. Yes b. No

6. Were you able to read the instructions well?
   a. Yes b. No

7. How easy was it to understand what to do / the instructions in each task?
   a. Extremely easy
   b. Very easy
   c. Somewhat easy
   d. Not so easy
   e. Not at all easy

8. Did you find the instructions for each task clear?
   a. Yes b. No

9. Did you experience any technical issues during your stay at the Smart Home?
   a. Yes b. No

10. Did you experience any issues with the sensors during your stay at the Smart Home?
    a. Yes b. No

11. Please note below your overall experience of the study

Easy

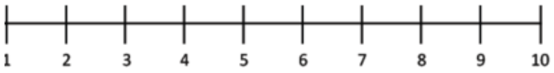

Difficult

12. Did you experience any issues during your stay at the Smart Home?
    a. Yes b. No

13. Did you experience any issues with the information provided to you during the study?
    a. Yes b. No

14. Did you understand the instructions in the Manual on how to perform the tasks?
    a. Yes b. No

15. Do you think any skills are required to interact with the proposed technologies?
    a. Yes b. No

16. Did you notice any issues with the position of the sensors?
    a. Yes b. No

17. Did you experience any issues during the overall study?
    a. Yes b. No

18. Did you feel any inconvenience during your stay at the Smart Home?
    a. Stress
    b. Depression
    c. Anxiety
    d. Frustration
    e. Nothing at all
    f. Other:

19. How would you describe your overall experience?
    a) Positive   b) Negative

20. Which task did you like the most?

21. Which task did you like the least?

22. Please note any other comment you would like to make regarding your stay at the SmartHome and your participation in the Tier 3 RADAR-AD study:

…………………………………………………………………………

1.3.3 Positive and Negative Affect

Please select from 1 (Very slightly or not at all) to 5 (Extremely) to refer to each one of the adjectives below:

Indicate the extent you have felt this way over the past week.

		Very slightly or not at all	A little	Moderately	Quite a bit	Extremely
1	Interested	1	2	3	4	5
2	Distressed	1	2	3	4	5
3	Excited	1	2	3	4	5
4	Upset	1	2	3	4	5
5	Strong	1	2	3	4	5
6	Guilty	1	2	3	4	5
7	Scared	1	2	3	4	5
8	Hostile	1	2	3	4	5
9	Enthusiastic	1	2	3	4	5
10	Proud	1	2	3	4	5
11	Irritable	1	2	3	4	5
12	Alert	1	2	3	4	5
13	Ashamed	1	2	3	4	5
14	Inspired	1	2	3	4	5
15	Nervous	1	2	3	4	5
16	Determined	1	2	3	4	5
17	Attentive	1	2	3	4	5
18	Jittery	1	2	3	4	5
19	Active	1	2	3	4	5
20	Afraid	1	2	3	4	5

Scoring:

Positive Affect Score: Add the scores on items 1, 3, 5, 9, 10, 12, 14, 16, 17, and 19. Scores can range from 10 – 50, with higher scores representing higher levels of positive affect.

Mean Scores: 33.3 (SD±7.2)

Negative Affect Score: Add the scores on items 2, 4, 6, 7, 8, 11, 13, 15, 18, and 20. Scores can range from 10 – 50, with lower scores representing lower levels of negative affect.

Mean Score: 17.4 (SD ± 6.2)

Your scores on the PANAS: Positive: Negative:

Watson, D., Clark, L. A., & Tellegen, A. (1988). Development and validation of brief measuresof positive and negative affect: the PANAS scales. Journal of personality and social psychology,54(6), 1063.

1.3.4 SUS Questionnaire

Please comment on the following statements by choosing your answer from 1-Strongly Disagree to 5-Strongly Agree

1. I think that I would like to use this system frequently
2. I found the system unnecessarily complex
3. I thought the system was easy to use
4. I think that I would need the support of a technical person to be able to use this system
5. I found the various functions in this system were well integrated
6. I thought there was too much inconsistency in this system
7. I would imagine that most people would learn to use this system very quickly
8. I found the system very cumbersome to use
9. I felt very confident using the system
10. I needed to learn a lot of things before I could get going with this system

Brooke, J B (2013). SUS – a retrospective. Journal of Usability Studies, Vol. 8, Issue 2, February 2013 pp. 29-40.

Brooke, J. (1986). "SUS: a "quick and dirty" usability scale". In P. W. Jordan, B. Thomas, B. A. Weerdmeester, & A. L. McClelland (eds.). Usability Evaluation in Industry. London: Taylor and Francis.
